# A compact and versatile tender X-ray single-shot spectrometer for online XFEL diagnostics

**DOI:** 10.1107/S1600577517012796

**Published:** 2018-01-01

**Authors:** Jens Rehanek, Christopher J. Milne, Jakub Szlachetko, Joanna Czapla-Masztafiak, Jörg Schneider, Thomas Huthwelker, Camelia N. Borca, Reto Wetter, Luc Patthey, Pavle Juranić

**Affiliations:** a Paul Scherrer Institut, Villigen-PSI 5232, Switzerland; b Institute of Physics, Jan Kochanowski University, Świętokrzyska 21, Kielce 25-406, Poland; c Institute of Nuclear Physics, Polish Academy of Sciences, PL-31342 Krakow, Poland

**Keywords:** free-electron laser, tender X-ray spectroscopy, single-shot measurement

## Abstract

One of the remaining challenges for accurate photon diagnostics at X-ray free-electron lasers (FELs) is the shot-to-shot, non-destructive, high-resolution characterization of the FEL pulse spectrum at photon energies between 2 keV and 4 keV, the so-called tender X-ray range. Here, a spectrometer setup is reported, based on the von Hamos geometry and using elastic scattering as a fingerprint of the FEL-generated spectrum. It is capable of pulse-to-pulse measurement of the spectrum with an energy resolution (Δ*E*/*E*) of 10^−4^, within a bandwidth of 2%. The Tender X-ray Single-Shot Spectrometer (TXS) will grant to experimental scientists the freedom to measure the spectrum in a single-shot measurement, keeping the transmitted beam undisturbed. It will enable single-shot reconstructions for easier and faster data analysis.

## Abstract 2

A single-shot spectrometer for the tender X-ray range is presented, based on the von Hamos geometry and using elastic scattering as a fingerprint of the XFEL-produced spectrum.

## 1. Introduction   

In a self-amplified spontaneous-emission free-electron laser (SASE FEL) the radiation spectrum is generated from a stochastic process, and varies from shot to shot (Emma *et al.*, 2004 ▸; Saldin *et al.*, 1998 ▸). Accurate evaluation of experimental results and the optimization of machine operation require that each photon pulse be measured with a high precision. In order to cover the shot-to-shot fluctuations, X-ray diagnostics tools are required that are capable of delivering spectral information reliably to a sufficiently high energy resolution as an online device. The coherence, high intensity, high brilliance and the small divergence of the generated FEL beam should be preserved during online measurement as much as possible. The quality of these properties could suffer from multiple reflections and scattering on many surfaces of numerous optical elements. To serve as an online device, spectrometric measurements need to be non-destructive to the beam, and it should, as a rule of thumb, have at least 90% or more transmission to the experiment.

While devices like the Paul Scherrer Institut (PSI) Photon Single-Shot Spectrometer (PSSS) (Rehanek *et al.*, 2017 ▸) have been developed to measure the photon spectrum generated by SwissFEL (Milne *et al.*, 2017 ▸) with an energy resolution (Δ*E*/*E*) of 10^−5^ in the hard X-ray range above 4 keV, no such photon diagnostics has been developed for the tender X-ray range, which is a photon energy range that is a specific focus of SwissFEL’s experimental station Alvra. The solution and the concept for the Tender X-ray Single-Shot Spectrometer (TXS) was found in the current devices based on curved crystals in Johann (1931 ▸), Johansson (1933 ▸) or in von Hamos (1933 ▸, 1934 ▸) geometries, which are designed to deliver information as spectrometers for resonant inelastic X-ray scattering (RIXS), X-ray emission spectroscopy (XES) or X-ray Raman scattering (XRS) studies at X-ray free-electron lasers, laboratories (Alonso-Mori *et al.*, 2012 ▸; Anklamm *et al.*, 2014 ▸), synchrotrons in the hard (Hayashi *et al.*, 2004 ▸) and tender X-ray ranges (Hoszowska & Dousse, 2004 ▸) and ion sources (Kavčič *et al.*, 2009 ▸, 2012 ▸). Our spectrometer concept is based on a dispersive von Hamos geometry and capable of measuring X-ray emissions over the entire photon energy range of interest. The spectrometer is combined with a scattering sample that has a low atomic number and low atomic density to collect the elastic (Rayleigh) scattering spectrum. This spectrum is an exact replica of the incoming FEL spectrum, as long as absorption edges are avoided in the scatterer, and the sample is chosen to be thin enough to allow the vast majority of the incoming light to proceed to the experiment unperturbed.

## 2. Experimental setup and theoretical estimations   

**Figure 1:**
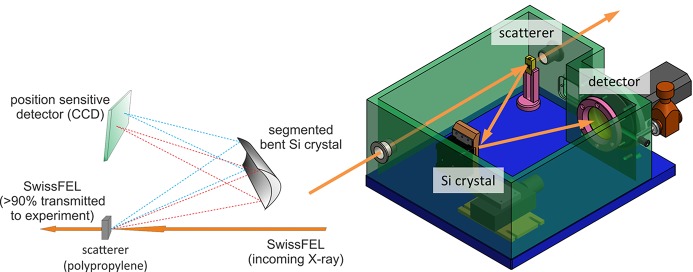
The principle of the von Hamos spectrometer setup in back-scattering configuration. Left: working principle; right: technical drawing.

The proposed setup has a single interaction point with the FEL-generated beam (see Fig. 1 ▸). The von Hamos spectrometer has been developed for RIXS measurements at PSI (Szlachetko *et al.*, 2012 ▸, 2013 ▸) as a tool for high-resolution spectroscopy of various samples at synchrotron and XFEL (Szlachetko *et al.*, 2014 ▸, 2016 ▸) radiation sources. The setup collects scattered photons over a large scattering angle onto an optical element composed of thin segmented Si crystals glued to a cylindrically bent substrate. The resolving power is determined by several factors, including the choice of the Si Miller indices, the geometry of the spectrometer and the detector pixel size; for further details, see Szlachetko *et al.* (2012 ▸, 2017 ▸).

The most efficient orientation of the von Hamos setup in this energy range is in the backward scattering direction to generate sufficient signal of the elastically scattered X-rays for the FEL spectrum measurement, which agrees with Sun *et al.* (2015 ▸). Further support for this choice of geometry is found in the atomic form factor tables of Hubbell *et al.* (1975 ▸), and the formulas for the elastic scattering of polarized light of Sun *et al.* (2015 ▸). We are using a partial cross section only [due to incoherent scattering, differential Klein–Nishina (Hubbell *et al.*, 1975 ▸)],
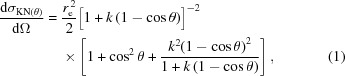
and calculating the scattered photons on this base and the coherent (Rayleigh) scattering cross section (Hubbell *et al.*, 1975 ▸),
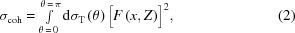
with the differential Thomson scattering cross section (Hubbell *et al.*, 1975 ▸)
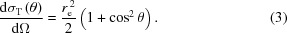
The coherent scattering does not play a very significant role in the case of our geometry, as the predominant amount of Rayleigh scattering occurs in the direction of the incoming light, forward and backwards, with a relatively small amount towards the sides (we are collecting at 67°). The transmitted light is based on the total cross section for absorption, which we obtain from Henke *et al.* (1993 ▸; CXRO, http://cxro.lbl.gov/), see Fig. 3. The treatment presented here ignores any effects of beam polarization, as equation (1) refers to non-polarized light. As all data were taken in the plane of the polarized light, equation (1) overestimates the cross section slightly (Hanson, 1988 ▸). For Swiss FEL instrumentation, it will be most efficient to build the von Hamos in a vertical scattering geometry, *i.e.* perpendicular to the direction of polarization, as this will allow for the maximum elastic X-ray scattering (Hanson, 1988 ▸).

**Figure 2:**
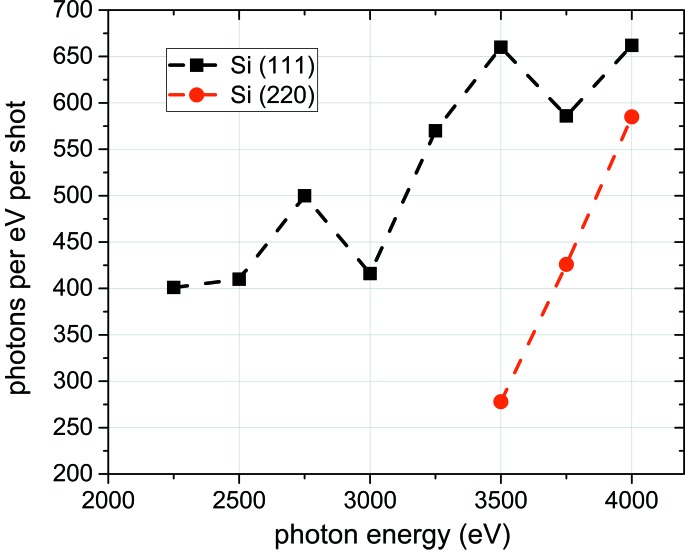
Expected signal rates *versus* photon energy for polypropyl­ene scatterer at an incoming photon flux of 10^12^ photons pulse^−1^ [black squares represent the calculation using the Si (111) reflection; red circles represent the calculation using the Si (220) reflection].

The attenuation by passing through the scattering sample follows simply 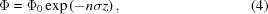
with Φ being the transmitted flux, Φ_0_ the incoming flux, σ the total cross section, *n* the atomic density and *z* the transmission length through the polypropyl­ene foil. We are concentrating on the incoherent back-scattered photon flux from the 4 µm foil. Since the TXS collects only the photons which are arriving at the bent crystal in the cone of the rocking curve around the Bragg reflection, the solid angle is narrow for the resolution limit that the device reaches. Simulations, based on the formulas and tables of Hubbell *et al.* (1975 ▸), show that the von Hamos spectrometer setup (perpendicular to the beam direction) would yield the photons per eV per shot shown in Fig. 2 ▸. The scattering sample in that case is polypropyl­ene, the spectrometer crystal assumed was Si (111) and the incident FEL flux was assumed to be 10^12^ photons pulse^−1^.

**Figure 3:**
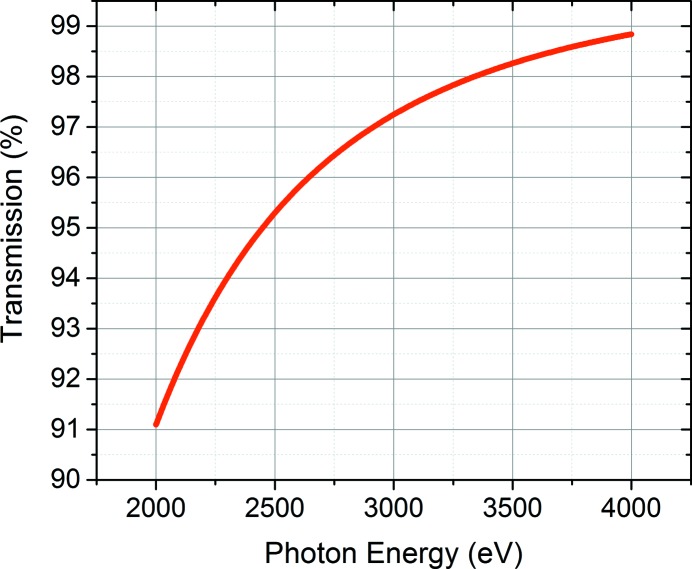
Calculated transmission of photons through 4 µm-thick polypropyl­ene. Data taken from CXRO.

The transmission of the sample is shown in Fig. 3 ▸. This estimate was made for a polypropyl­ene film, which has a transmission between 91% at 2 keV and 99% at 4 keV.

## 3. Experimental results and discussion   

The test experiment was performed at the PHOENIX I (X07MA/B) beamline at the Swiss Light Source (Böge, 2002 ▸; Streun, 2016 ▸), Paul Scherrer Institute, Villigen, Switzerland. The beamline generates photons in the energy range 0.8–8 keV using a double-crystal monochromator. For the experiments presented here a silicon (111) crystal was used. The optical concept follows that of the LUCIA beamline (Flank *et al.*, 2006 ▸).

**Figure 4:**
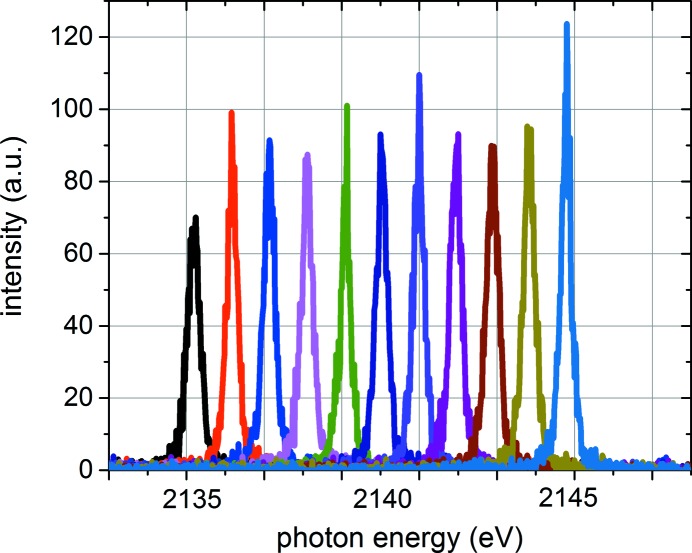
Scan over a 0.5% bandwidth at 2140 eV. Acquisition time: 10 s at each point; scanning step size: 1 eV. Curves are not normalized.

As a proof of principle, we focused on the performance of the setup at two different photon energies within the tender X-ray range, 2140 eV [around the P *K*_β_ emission, using the Si (111) reflection] and 3495 eV [using the Si (220) reflection]. The corresponding Bragg angles on the crystal were around 67°. Because of the back-scattering geometry, the scattering angle from the sample (defined as the angle between the incident and outgoing beams) equals 90° minus the Bragg angle. The X-rays diffracted by the crystal were then recorded with a back-illuminated type charge-coupled device (CCD) with a pixel size of 25 µm. The CCD has around 90–95% detection efficiency at the measured X-ray energies. The unfocused beam spot size on the sample was 50 µm in the dispersive direction and 250 µm along the focusing axis. Fig. 4 ▸ shows the results from scanning the photon energy around 2140 eV in steps of 1 eV, using the 4 µm polypropyl­ene foil as elastic scattering sample at an incoming flux of 1.14 × 10^11^ photons s^−1^ (0.015% bandwidth)^−1^. The acquisition time was 10 s at each point, in order to simulate the number of photons coming in as expected from SwissFEL.

At the photon energy of 3495 eV we made use of the Si (220) reflection. We cross-checked the scattering efficiency and resulting measured signal-to-noise for two different materials, polypropyl­ene (C_3_H_6_) and Kapton (polyimide, C_22_H_10_N_2_O_5_). The results of the data evaluation are summarized in Table 1 ▸. These results agree to within 90% with the theoretically calculated values, using Si (111) and Si (220) reflections, respectively. The number of photons arriving at the detector is sufficient for the accuracy we need for the detector. The increase of intensity towards higher photon energies in the recorded data is a result of different levels of filtering noise during evaluation and different sensitivity of the detector pixels to different photon energies. This will be taken into consideration and the measured signal will be corrected accordingly.

## 4. Summary and outlook   

We have shown a simple and versatile setup for measuring SwissFEL-generated tender X-ray radiation on a single-shot basis with an energy resolution of ∼10^−4^, within a bandwidth of 2%. This spectrometer could be used as a mobile device for photon-in/photon-out experiments at XFELs or an application at synchrotron sources. In case polypropyl­ene turns out to be ill-fitting for an application, one could easily make use of other scattering materials, such as Kapton (polyimide). The experiment agrees very well with the theoretical estimations made beforehand, which enables us to continue with the development and building the Tender X-ray Single-Shot Spectrometer for SwissFEL.

## Figures and Tables

**Table 1:** Experimental results, using 4 µm scattering sample of C_3_H_6_

	Photon energy	
	2140 eV	3495 eV
Incoming (photons s^−1^)	1.14 × 10^11^	6.7 × 10^10^
FWHM (eV)	0.4 (19 pixels)	0.47 (19 pixels)
Δ*E*/*E*	1.9 × 10^−4^	1.4 × 10^−4^
Possible full range (eV)	±21.3 (2% bandwidth)	±34 (2% bandwidth)
No. of photons calculated at 3495 eV, reflected in Darwin width around Bragg angle†		277
No. of photons measured at detector†, within bandwidth of 0.015% (0.5 eV) at 3495 eV		290

† After 10 s integration time.
